# Clinical features of Guillain–Barré syndrome patients with elevated serum creatine kinase levels

**DOI:** 10.1186/s12883-020-01796-z

**Published:** 2020-05-27

**Authors:** Takafumi Hosokawa, Hideto Nakajima, Taiki Sawai, Yoshitsugu Nakamura, Eri Sano, Akihiro Tsukahara, Kiichi Unoda, Shimon Ishida, Sadaki Sakane, Fumiharu Kimura, Shigeki Arawaka

**Affiliations:** 1 Division of Neurology, Department of Internal Medicine IV, Osaka Medical College, 2-7 Daigaku-machi, Takatsuki, Osaka, 569-8686 Japan; 2 Department of Internal Medicine, Hirakata City Hospital, 2-14-1 Kinya-honnmachi, Hirakata, Osaka, 573-1013 Japan; 3 Division of Neurology, Department of Medicine, Nihon University School of Medicine, 30-1 Oyaguchi Kami-cho, Itabashi-ku, Tokyo, 173-8610 Japan

**Keywords:** Creatine kinase, Guillain-Barré syndrome, AIDP, AMAN, Reversible conduction failure

## Abstract

**Background:**

It is not well defined whether Guillain–Barré syndrome (GBS) patients with elevated serum creatine kinase (CK) levels have characteristic clinical features and are related to the subgroups of GBS.

**Methods:**

We retrospectively studied 51 consecutive patients with GBS, who visited our hospital, and compared clinical, laboratory and electrophysiological findings between patients with and without elevated CK levels.

**Results:**

Of 51 patients, 14 patients (27%) showed an elevation of serum CK levels. When compared with patients with the normal CK levels, the ratios of male, antecedent infections, and anti-GM1 antibody positivity were significantly higher in patients with elevated CK levels. The ratios of hypoesthesia, cranial nerve involvement, and urinary retention were significantly less in patients with elevated CK levels. There were no significant differences in disability at peak between two groups. In the electrophysiological examination, sensory nerve abnormalities were not observed. Although some patients with elevated CK levels showed prolongation of distal motor latencies (DMLs) and increase of durations in the initial examination, development of the prolongation of DMLs and increase of durations was not observed in the follow-up examinations. The findings were consistent with acute motor axonal neuropathy (AMAN) with reversible conduction failure (RCF) but not acute inflammatory demyelinating polyneuropathy (AIDP).

**Conclusions:**

The results suggest that the GBS patients with elevated CK levels represent not a group of AIDP but a group of AMAN with axonal degeneration or RCF even though the initial electrophysiological examination shows AIDP pattern.

## Introduction

Guillain–Barré syndrome (GBS) is classified into two major subgroups: acute motor axonal neuropathy (AMAN) and acute inflammatory demyelinating polyneuropathy (AIDP) [1–6]. AMAN is further classified into the subgroups, such as AMAN with axonal degeneration and reversible conduction failure (RCF) [7]. AMAN with RCF is characterized clinically by rapid improvement [5, 8, 9]. It is important to accurately diagnose the subgroups because each subgroup has distinct clinical, pathological, and immunological features.

While cerebrospinal fluid test abnormalities in Guillain–Barré syndrome are well known, serum test abnormalities have also been reported. One such serum abnormality in GBS is an elevation in creatine kinase (CK) levels [10]; however, it is unclear whether GBS with elevated CK levels has homogeneous features, and if so, what those features are. Especially, association of GBS with elevated CK levels and GBS subgroup is unclear. One of the reasons may be the difficulty and confusion to clinically diagnose GBS subgroups including AMAN with RCF by using a single electrophysiological examination and conventional criteria of classification. However, previous reports demonstrated that the follow-up electrophysiological examinations are useful for resolving this issue [11–13]. Moreover, focusing on the presence or absence of sensory nerve conduction abnormalities, we established new system classifying GBS into AMAN with axonal degeneration, AMAN with RCF, and true AIDP by using only a single electrophysiological examination.

In this study, we focused on the GBS patients with elevated CK levels. We investigated whether these patients are related to the specific subgroups of GBS by using serial electrophysiological studies and our system classifying GBS into subgroups.

## Material and methods

### Patients

We retrospectively evaluated 51 consecutive GBS patients who visited Osaka Medical College Hospital from January 2005 to December 2016 (31 men, 20 women; mean age 45.9 years, range 17–80 years). These patients underwent initial nerve conduction examinations within 17 days of onset. All patients fulfilled the clinical criteria for GBS [14], except the point regarding areflexia or hyporeflexia. We enrolled these patients, because the presence of GBS patients with normal or exaggerated tendon reflexes was shown previously [8, 15]. Three GBS patients were excluded from this study, because these patients had diabetic neuropathy and it was difficult to appropriately separate abnormalities in the sensory nerve conduction by GBS from those by diabetic neuropathy.

Patient disabilities were evaluated according to the Hughes disability grade scale as follows: grade 0, healthy; grade 1, minor signs and symptoms, able to run; grade 2, able to walk independently; grade 3, able to walk with a walker or support; grade 4, bed or chair bound; grade 5, assisted respiration required for at least part of the day; and grade 6, dead [16]. Elevation of serum CK levels was defined as the values > 200 U/L (normal range 30–200 U/L) for more than 3 days during 4 weeks after onset. The reason why we required more than 3 days as the duration of CK elevation is to exclude the influence of non-pathogenic conditions. Although non-pathogenic conditions such as physical exercise are known to cause temporal CK elevation, a large community study found that repeat CK levels in people with incidentally discovered CK elevation were normal after rest of 3 days in 70% of cases [17]. The reason why determination of CK elevation was based on CK levels during 4 weeks after onset is to minimize bias caused by different observation time, as almost all patients underwent laboratory tests during 4 weeks after onset at least. CK levels were measured before electromyographic examination, if it was performed.

### Electrophysiological examinations

Motor and sensory nerve conduction examinations were performed using MEB-9104 Neuropack mu® (Nihon Kohden, Japan) according to the methods and reference values as described by Kimura et al. [18] Motor nerve conduction examinations were performed on the median, ulnar, peroneal, and tibial nerves. Compound action potential (CMAP) duration was defined as the time from the onset of the initial negative phase until the last negative deflection of CMAP returned to the baseline. Antidromic sensory nerve conduction examinations were performed on the median, ulnar, and sural nerves. Based on the results of initial electrophysiological examinations, patients were classified into the AIDP or AMAN pattern according to the criteria of Ho et al. [1] Although their criteria set includes unequivocal temporal dispersion for the detection of demyelination, how much temporal dispersion of CMAP should be considered as “unequivocal” one is not defined in the criteria. Therefore, we used a distal CMAP duration > 6.6 ms in the median, > 6.7 ms in the ulnar, > 7.6 ms in the peroneal, and > 8.8 ms in the tibial nerves [19] or > 30% increase in duration ratio of the proximal CMAP to distal one in all nerves [20]. When the electrophysiological findings did not fulfill the criteria for AIDP or AMAN patterns, the patients were designated as unclassified GBS. The absence of F waves was defined as the absence or marked decrease (persistence < 20%) of F waves in at least two nerves [21]. Patients were considered to have sensory nerve conduction abnormalities when the sensory nerve action potential amplitude was < 50% of the lower limit of normal range in at least two nerves [22]. In this study, because the electrodiagnosis and disease entity may not be equivalent, the electrodiagnosis of AIDP and AMAN were respectively expressed as “AIDP pattern” and “AMAN pattern,” and the disease entity as AIDP and AMAN (AMAN with axonal degeneration and AMAN with RCF) from now on.

### Definitions of GBS subgeigroups

In accordance with our previous findings [23, 24], AIDP was defined as electrodiagnosis of “AIDP pattern” based on Ho’s criteria with sensory nerve conduction abnormalities. AMAN with axonal degeneration was defined as electrodiagnosis of “AMAN pattern.” AMAN with RCF was defined as electrodiagnosis of “AIDP pattern” without sensory nerve conduction abnormalities or unclassified.

### Statistical analyses

Differences in mean values between two groups were analyzed using the Mann–Whitney U test, and differences in frequencies were analyzed using the Fisher exact probability test. Time to event was analyzed by the Kaplan-Meier method and the log-rank test for trend. Statistical significance was set at *P* < 0.05. All analyses were performed using GraphPad PRISM version 5.01 (GraphPad Software, San Diego, CA, USA).

## Results

### Clinical and laboratory features of GBS patients with elevated CK levels

Table 1 shows a comparison of clinical and laboratory findings of GBS patients with and without elevated CK levels. Of 51 patients, 14 (27%) patients showed an elevation of serum CK levels and 37 (73%) patients showed normal CK levels. The ratio of male in patients with elevated CK levels (*n* = 12) were significantly higher than that of patients with normal CK levels (*n* = 19, *P* = 0.029). The incidence of infections prior to the onset of GBS in patients with elevated CK levels (*n* = 13) were significantly higher than that of patients with normal CK levels (*n* = 22, *P* = 0.039). As antecedent infections, the ratio of upper respiratory tract infection (URTI) in patients with elevated CK levels (*n* = 8) were higher than that of patients with normal CK levels (*n* = 8, *P* = 0.021). Anti-GM1 antibody was measured in 12 and 32 patients with and without elevated CK levels, respectively. The positive ratio of anti-GM1 antibody in patients with elevated CK levels (*n* = 8) were higher than that in patients with normal CK levels (*n* = 8, *P* = 0.016). The incidences of hypoesthesia and cranial nerve involvement in patients with elevated CK levels (*n* = 1 and 2, respectively) were significantly less than those in patients with normal CK levels (*n* = 15 and 19, respectively, *P* = 0.016). Patients with elevated CK levels showed no urinary retention. There were no significant differences in disability at peak between two groups. Moreover, there were no significant differences in time to Hugh grade 1 between two groups (data was not shown).

**Table 1:** Clinical and laboratory features of 51 patients with GBS

	GBS patients with elevated CK levels (*n* = 14)	GBS patients with normal CK levels (*n* = 37)	*P*-value
Age, years, mean ± SD	41.2 ± 10.2	47.7 ± 21.1	NS
Males, n (%)	12 (86)	19 (51)	0.029
Antecedent infection, n (%)	13 (93)	22 (59)	0.039
Gastroenteritis, n (%)	5 (35)	14 (38)	NS
Upper respiratory tract infection, n (%)	8 (57)	8 (21)	0.021
Clinical symptoms			
Hypoesthesia, n (%)	1 (7)	15 (41)	0.039
Cranial nerve involvement, n (%)	2 (14)	19 (51)	0.025
Ophthalmoparesis	1 (7)	7 (19)	NS
Facial palsy	1 (7)	10 (27)	NS
Oropharyngeal palsy	2 (14)	17 (46)	NS
Urinary retention, n (%)	0 (0)	12 (32)	0.022
Preserved tendon reflexes, n (%)	9 (64)	16 (43)	NS
Anti-GM1 antibody, n (%)	8/12 (67)	8/32 (25)	0.016
Hughes grade at peak			NS
1	1 (7)	3 (8)	
2	7 (50)	13 (35)	
3	2 (14)	4 (11)	
4	3 (21)	13 (35)	
5	1 (7)	3 (8)	
6	0 (0)	1 (3)	

*Abbreviations*: *GBS* Guillain–Barré syndrome, *CK* Creatine kinase, *NS* Not significant

### The electrophysiological features and electrodiagnosis by Ho’s criteria at the initial examinations

At the initial electrophysiological findings, 51 patients were classified as having the “AIDP pattern” (*n* = 30, 59%), the “AMAN pattern” (*n* = 8, 16%), and electrophysiologically unclassified (*n* = 13, 25%), based on Ho’s criteria. One patient with the “AMAN pattern” with normal CK levels showed sensory nerve conduction abnormalities, indicating the “acute motor and sensory axonal neuropathy pattern” in the strict sense [25]. Table 2 shows findings in the initial electrophysiological examinations. Although, in patients with elevated CK levels, one patient with the “AMAN pattern” showed hypoesthesia, electrophysiological criteria of sensory nerve abnormality were not fulfilled. There were no GBS patients with elevated CK levels clearly having sensory nerve conduction abnormalities.

**Table 2:** Comparison of electrophysiological findings and electrodiagnosis by Ho’s criteria at the initial examination between GBS patients with and without elevated CK levels

	GBS patients with elevated CK levels (*n* = 14)	GBS patients with normal CK levels (*n* = 37)	*P*-value
Electrodiagnosis			
AIDP pattern	8 (57)	22 (59)	NS
AMAN pattern	3 (21)	5 (14)	NS
Unclassified	3 (21)	10 (27)	NS
Sensory nerve conduction abnormality	0 (0)	16 (43)	0.002
Absence of F waves	5 (35)	13 (35)	NS

*Abbreviations*: *GBS* Guillain–Barré syndrome, *CK* Creatine kinase, *AIDP* Acute inflammatory demyelinating polyneuropathy, *AMAN* Acute motor axonal neuropathy, *NS* Not significant

### GBS subgroups by our definitions

Table 3 shows subgroups of GBS patients with and without elevated CK levels, based on our definitions. All GBS patients with elevated CK levels were diagnosed as AMAN with axonal degeneration or RCF. In contrast, > 40% of GBS patients with normal CK levels were diagnosed as AIDP. The ratio of AMAN following URTI in patients with elevated CK levels (*n* = 8, 2 with axonal degeneration and 6 with RCF) were significantly higher than that of patients with normal CK levels (*n* = 4, 2 with axonal degeneration and 2 with RCF, *P* = 0.0014). Of patients with AMAN following URTI, 5 patients with elevated CK levels (2 with axonal degeneration and 3 with RCF) and 1 patient with normal CK levels (1 with RCF) were positive for anti-GM1 antibody. Table 4 shows serum CK levels and clinical course of 14 patients with elevated CK levels. To anonymize the identifying information, specific ages were grouped into age ranges. None of the 14 patients with elevated CK levels were taking statin. CK values were not significantly correlated with disability grades or CMAP amplitudes. In most of GBS patients with elevated CK levels, elevation of CK began within 2 weeks after onset, and CK levels peaked within 2 weeks after nadir of disability.

**Table 3:** Comparison of subgroups by our definitions between GBS patients with and without elevated CK levels

	GBS patients with elevated CK levels (*n* = 14)	GBS patients with normal CK levels (*n* = 37)	*P*-value
AIDP	0 (0)	15 (41)	0.005
AMAN	14 (100)	22 (59)	0.005
AMAN with axonal degeneration	3 (21)	5 (14)	NS
AMAN with RCF	11 (79)	17 (46)	NS

*Abbreviations*: *GBS* Guillain–Barré syndrome, *CK* Creatine kinase, *AIDP* Acute inflammatory demyelinating polyneuropathy, *AMAN* Acute motor axonal neuropathy, *RCF* Reversible conduction failure, *NS* Not significant

**Table 4:** CK levels and clinical features of 14 patients with Guillain–Barré syndrome with elevated CK levels

Patient No.	Age/sex	CK			Cranial nerve involvement	Limb weakness			CMAP amplitude (mV) Right median nerve	Treatment	Disability			
		Time until first elevation (days)	Time until peak (days)	Level at peak (U/L)		Arm or leg dominant	Proximal or distal dominant	Symmetric or asymmetric			Time until nadir (days)	At nadir (HG)	14 days after onset (HG)	28 days after onset (HG)
1	20–29/M	4	4	1937	None	Arm dominant	Distal dominant	Symmetric	0.5	IVIG	4	2	1	1
2	30–39/M	9	9	1264	None	No dominance	No dominance	Symmetric	6.2	IVIG	2	3	2	1
3	30–39/M	11	11	795	None	Arm dominant	Distal dominant	Symmetric	3.2	IVIG	11	2	2	1
4	20–29/M	16	24	591	None	No dominance	No dominance	Symmetric	7.1	IVIG	15	2	2	1
5	30–39/M	11	14	550	Ophthalmoplegia, Facial weakness, bulbar weakness	No dominance	Distal dominant	Symmetric	7	IVIG + steroid pulse	4	4	2	2
6	40–49/M	2	10	515	None	Arm dominant	Distal dominant	Symmetric	2.5	IVIG	4	2	2	2
7	30–39/M	9	9	433	None	Arm dominant	Distal dominant	Symmetric	5.2	None	6	2	2	0
8	30–39/M	25	28	429	Bulbar weakness	Leg dominant	No dominance	Symmetric	0.5	IVIG + plasma exchange	10	5	5	5
9	50–59/M	7	7	402	None	Arm dominant	No dominance	Asymmetric	7.5	IVIG	12	1	1	1
10	50–59/M	2	4	396	None	No dominance	Distal dominant	Asymmetric	7	IVIG	5	4	1	1
11	40–49/F	6	10	351	None	No dominance	No dominance	Symmetric	7.8	IVIG	8	2	1	1
12	40–49/M	3	5	319	None	No dominance	No dominance	Symmetric	8.8	IVIG	4	2	2	1
13	40–49/M	10	16	300	None	No dominance	No dominance	Symmetric	2.5	IVIG	4	4	3	2
14	60–69/F	5	5	288	None	No dominance	Distal dominant	Symmetric	7.5	IVIG	4	3	2	1

To anonymize the identifying information, specific ages were grouped into age ranges

*Abbreviations*: *CK* Creatine kinase, *CMAP* Compound action potential, *HG* Hugh grade, *IVIG* Intravenous immunoglobulin

### The electrophysiological features at the follow-up examinations

**Fig. 1:**
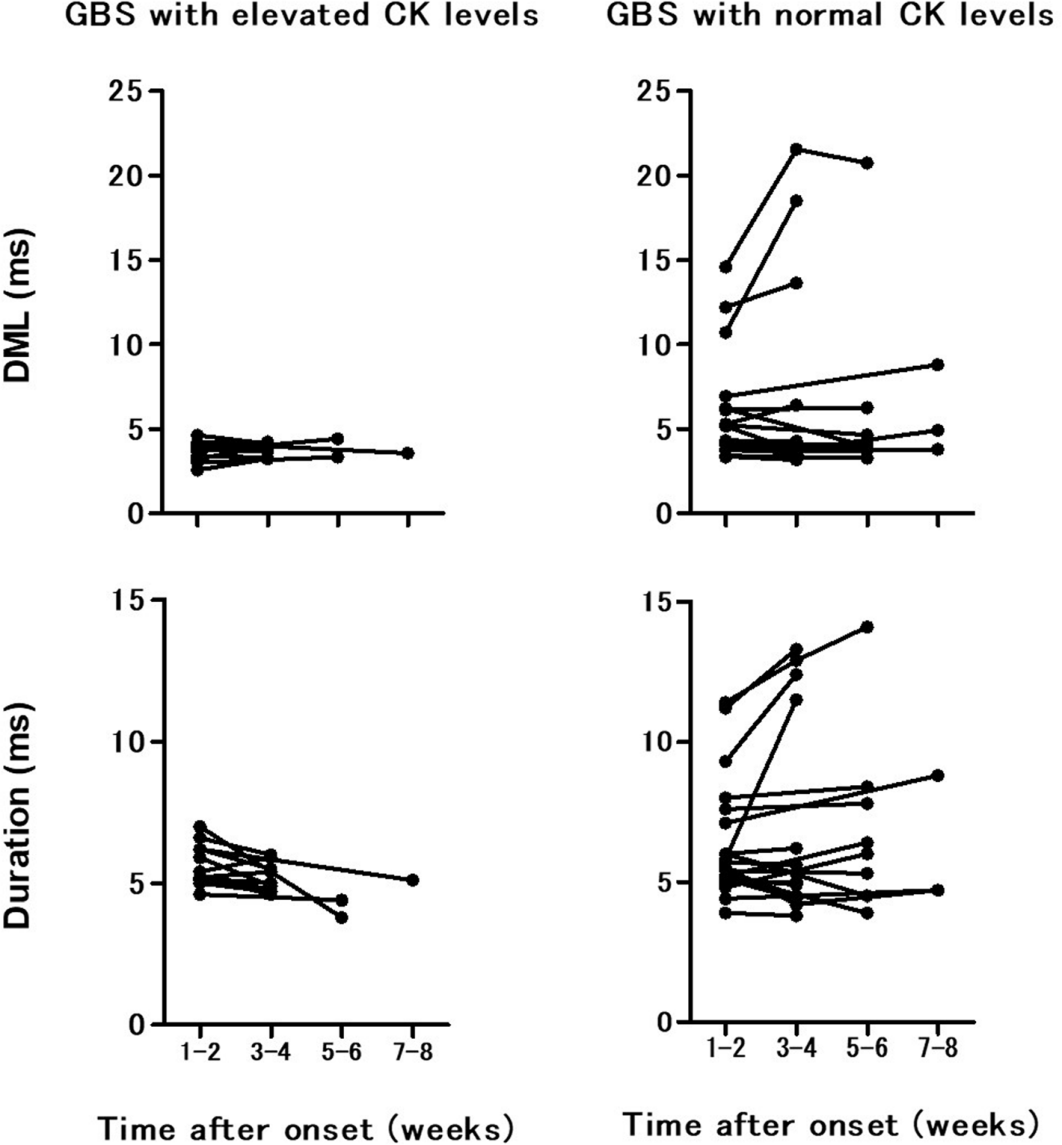
Serial findings of DML and duration in the right median motor nerve of GBS patients with and without elevated CK levels. GBS, Guillain–Barré syndrome; CK, creatine kinase; DML, distal motor latency

Of 14 patients with elevated CK levels, 12 patients underwent follow-up electrophysiological examinations. Of 37 patients with normal CK levels, 22 patients underwent follow-up electrophysiological examinations. Figure 1 shows temporal changes of electrophysiological parameters at the right median nerve. Some GBS patients with elevated CK levels showed prolongation of distal motor latencies (DMLs) and increase of durations in the initial examinations. However, none showed development of increased durations and prolonged DMLs in the follow-up examinations, while some GBS patients with normal CK levels did show development.

## Discussion

The present study shows that the elevation of CK levels is seen in GBS. 27% of patients had the elevation of CK levels in the first 4 weeks after onset. In this study, although conventional classification using a single electrophysiological examination and Ho’s criteria did not indicate any relationship between GBS patients with elevated CK levels and specific electrophysiological patterns of GBS, our classification indicates that all GBS patients with elevated CK levels represent a group of AMAN with axonal degeneration or RCF. Electrodiagnosis based on conventional classification system is reported to be often inconsistent with the true subgroup of GBS. 11–41% of patients with GBS failed to judge AIDP or AMAN, because electrophysiological findings did not fulfill the criteria for “AIDP pattern” or “AMAN pattern.” [1, 11, 12, 26] Additionally, AMAN with RCF is misdiagnosed as AIDP, because electrophysiological examinations at the acute phase indicate the “AIDP pattern,” such as prolongation of DMLs and increase of durations [11, 26, 27]. This means that the “AIDP pattern” on electrophysiological examinations reflects two different subgroups, AIDP and AMAN with RCF. However, we previously reported that AMAN with RCF did not show sensory nerve conduction abnormalities in contrast to true AIDP [23]. We also demonstrated that the true disease subgroup of “AMAN pattern” and unclassified GBS is AMAN with axonal degeneration and AMAN with RCF, respectively [23, 24]. Using these findings, we established the new system classifying GBS into AMAN with axonal degeneration, AMAN with RCF, and true AIDP. In this study, our classification system with these improvements made it possible to discover new findings about GBS subgroups, which conventional classification system could not discover.

While a limited number of patients underwent serial electrophysiological examinations, serial examinations also confirm that GBS with elevated CK levels is AMAN with axonal degeneration or RCF, not a group of AIDP. In the initial electrophysiological examinations, some GBS patients with elevated CK levels were classified as the “AIDP pattern” based on Ho’s criteria, which showed prolongation of DMLs and increase in durations. However, they showed small extent of demyelinating features, which is consistent with RCF in the first place [11, 26, 27]. Notably, in the follow-up examination, they showed electrophysiological changes consistent with AMAN with RCF, such as rapid normalization of increased duration [7, 8, 11, 12, 26, 27]. That is, none of GBS patients with elevated CK levels showed changes consistent with true AIDP, such as the development of this prolongation of DMLs and increase of durations [28, 29]. On the other hand, it was seen in some GBS patients with normal CK levels, suggesting that GBS with normal CK levels includes true AIDP.

Several features of GBS patients with elevated CK levels other than electrophysiological findings also confirm that GBS patients with elevated CK levels represent a group of AMAN. In our study, patients with elevated CK levels frequently had antecedent infections and anti-GM1 antibody, rarely had hypoesthesia and cranial nerve involvement, and did not have urinary retention as signs of autonomic failure; all of the features were consistent with AMAN and not true AIDP [1, 3–5]. Conversely, while the most common antecedent infection in AMAN was gastroenteritis, particularly from *Campylobacter jejuni*, the most common antecedent infection in GBS patients with elevated CK levels in this study was URTI. Moreover, in some GBS patients with elevated CK levels and anti-GM1 antibody, the antecedent infection was URTI even though anti-GM1 antibody is usually related not with URTI but with gastroenteritis such as C.jejuni infection. AMAN following URTI, including with anti-GM1 antibody, may be a specific feature of GBS patients with elevated CK levels.

To our knowledge, while we found some case reports of GBS patients with elevated CK levels [10, 30, 31], there is only one report of a series of GBS patients in whom CK levels was investigated [32]. That report showed a 52% incidence of the elevation of CK levels in GBS and pain associated with the elevation of CK levels in GBS patients; however, the authors did not record the disease subgroup of GBS and consequently did not mention the correlation between GBS disease subgroup and the elevation of CK levels. Therefore, our study is the first study of a series of GBS patients in whom correlation between GBS disease subgroup and elevation of CK levels is investigated, demonstrating that GBS patients with elevated CK levels represent a group of AMAN.

Although the mechanism of elevation of CK levels in GBS is still uncertain, several possible mechanisms were proposed in the literature. One possible mechanism is associated with rhabdomyolysis caused by antecedent infection [30]. This mechanism, however, is unlikely because the patients with rhabdomyolysis showed markedly raised CK levels, which were not consistent with our cases with moderate raised CK levels. Another possible mechanism is that rapid denervation due to axonal damage can result in the release of muscle enzymes [10]. It is also proposed that CK elevation could be caused by painful muscle cramp associated with active denervation on the basis of the finding that most of GBS patients with elevated CK levels developed painful sensation. Regrettably, we could not investigate the association between elevation of CK levels and pain, because of retrospective nature of our study. However, the mechanism that denervation could cause CK elevation is consistent with our observation that elevation of CK levels occurred in AMAN with RCF as well as AMAN with axonal degeneration but not in AIDP, because the denervation could occur in AMAN with RCF as well as AMAN with axonal degeneration but not in demyelination alone [33, 34]. The mechanism could explain our observations that elevation of CK began within 2 weeks after onset and that peak of CK followed nadir of disability within 2 weeks, because denervation follows axonal degeneration within at most 2 weeks in proportion to the distance of the muscle from the injury site. The mechanism could also explain that in some of our patients, the elevation of CK levels occurred within a few days, because motor nerve terminal located a bit distant from muscle is preferentially affected at first in GBS. Moreover, given that not all AMAN patients showed CK elevation, a specific mechanism other than those directly caused by axonal damage may also be necessary for CK elevation. Mentioned above, we found that AMAN following URTI, including with anti-GM1 antibody, may be a specific feature of GBS patients with elevated CK levels. Although we could not investigate the antecedent pathogens, AMAN following URTI, including with anti-GM1 antibody, was reportedly associated with a specific pathogen such as *Haemophilus influenzae* [35]. Therefore, the specific pathogen not identified in this study may elicit not only URTI and subsequent AMAN but also additional mechanisms that are specific to elevation of CK levels.

Our study has several limitations. First, it is small and retrospective and includes only patients from Japan, where AMAN patients are more frequent than other Western countries. Further large prospective studies in various population groups are needed. Second, although serial electrophysiological examinations were performed, the number of nerves, timing, frequency, and period varied for each patient. In some patients, serial examinations were performed only a few times over a short period or not performed at all. In motor nerves, nerve conduction examination of the right median nerve was most often performed in our study. Therefore, we only showed the results of nerve conduction examinations on the right median nerve. Further studies with more frequent electrophysiological assessment for more and predetermined nerves at predetermined time points over a longer time period could identify these features in greater detail and with greater confirmation. Third, as mentioned above, while pathogens of antecedent infections are associated with various clinical phenotypes in GBS, we did not perform the comprehensive test to identify pathogens of antecedent infection, such as serum antibody to *C. jejuni*. Fourth, the test for anti-ganglioside antibodies was performed; however, only antibodies against GM1 and GQ1b were assessed in a limited number of patients. Information about antibodies to a ganglioside complex consisting of GM1 and GalNAc-GD1a is especially important as these antibodies have been reported to be associated with a pure motor variant with RCF following respiratory infection [36–38]. Information about IgG subclass (IgG1 or IgG3) of anti-GM1 antibodies is also important because the IgG subclasses of AMAN-associated antibodies have been reported to be associated with antecedent infection and the speed of recovery of GBS [39]. However, we did not investigate these antibodies in this study.

## Conclusions

We showed that GBS patients with elevated CK levels represented a group of AMAN. Moreover, we showed that elevated CK tended to occur during the acute phase of AMAN following URTI. We also propose CK elevation as a candidate of the clinical features of GBS which differ between AMAN and AIDP. The presence of CK elevation could facilitate the accurate diagnosis of the disease subgroup.

## Data Availability

The datasets used and/or analyzed during the current study are available from the corresponding author on reasonable request.

## Abbreviations

AIDP: Acute inflammatory demyelinating polyneuropathy
AMAN: Acute motor axonal neuropathy
CK: Creatine kinase
CMAP: Compound action potential
DML: Distal motor latency
GBS: Guillain–Barré syndrome
IVIG: Intravenous immunoglobulin
RCF: Reversible conduction failure
URTI: Upper respiratory tract infection
